# Spliceosome mutations are associated with clinical response in a phase 1b/2 study of the PLK1 inhibitor onvansertib in combination with decitabine in relapsed or refractory acute myeloid leukemia

**DOI:** 10.1007/s00277-023-05442-9

**Published:** 2023-09-13

**Authors:** Peter J P Croucher, Maya Ridinger, Pamela S. Becker, Tara L. Lin, Sandra L. Silberman, Eunice S. Wang, Amer M. Zeidan

**Affiliations:** 1 Cardiff Oncology Inc., 11055 Flintkote Avenue, San Diego, CA 92121 USA; 2 Leukemia Division, Department of Hematology and Hematopoietic Cell Transplantation, City of Hope National Medical Center, Duarte, CA 91010 USA; 3 Division of Hematologic Malignancies and Cellular Therapeutics, University of Kansas, Kansas City, KS 66205 USA; 4 SLS Oncology LLC, Durham, NC 27713 USA; 5 Leukemia Service, Roswell Park Comprehensive Cancer Center, Buffalo, NY 14263 USA; 6 Yale University and Yale Cancer Center, New Haven, 333 Cedar Street, PO Box 208028, New Haven, CT 06520-8028 USA

**Keywords:** Splicing, Onvansertib, AML, Biomarker, Hypomethylating agents, PLK1

## Abstract

PLK1 is overexpressed in acute myeloid leukemia (AML). A phase 1b trial of the PLK1 inhibitor onvansertib (ONV) combined with decitabine (DAC) demonstrated initial safety and efficacy in patients with relapsed/refractory (R/R) AML. The current study aimed to identify molecular predictors of response to ONV + DAC in R/R AML patients. A total of 44 R/R AML patients were treated with ONV + DAC and considered evaluable for efficacy. Bone marrow (BM) samples were collected at baseline for genomic and transcriptomic analysis (*n* = 32). A 10-gene expression signature, predictive of response to ONV + DAC, was derived from the leading-edge genes of gene set enrichment analyses (GSEA). The gene signature was evaluated in independent datasets and used to identify associated mutated genes. Twenty percent of the patients achieved complete remission, with or without hematologic count recovery (CR/CRi), and 32% exhibited a ≥50% reduction in bone marrow blasts. Patients who responded to treatment had elevated mitochondrial function and OXPHOS. The gene signature was not associated with response to DAC alone in an independent dataset. By applying the signature to the BeatAML cohort (*n* = 399), we identified a positive association between predicted ONV + DAC response and mutations in splicing factors (SF). In the phase 1b/2 trial, patients with SF mutations (SRSF2, SF3B1) had a higher CR/CRi rate (50%) compared to those without SF mutations (9%). PLK1 inhibition with ONV in combination with DAC could be a potential therapy in R/R AML patients, particularly those with high OXPHOS gene expression and SF mutations.

**Supplementary Information:**

The online version contains supplementary material available at 10.1007/s00277-023-05442-9.

## Introduction

Acute myeloid leukemia (AML) is predominantly a disorder of older patients (median age at diagnosis: 69 years [1]) that is characterized by the clonal expansion of myeloid blasts and results in bone marrow failure. AML typically features epigenetic modifications, with mutations in genes involved in DNA methylation and histone modification [2, 3]. Consequently, AML patients who are unable to tolerate standard intensive induction chemotherapy have historically received hypomethylating agents (HMA) such as decitabine (DAC) and azacytidine (AZA), or alternatively low-dose cytarabine (LDAC). However, complete response rates are low and durations short [4]. The introduction of the BCL2 inhibitor venetoclax (VEN) in combination with HMA/LDAC has greatly improved frontline outcomes with response rates up to 67–73% and median OS up to 15–17.5 months [5, 6]. However, patients with relapsed or refractory (R/R) AML still have few effective treatment options and poor outcomes (median OS: 3–7 months) [6, 7].

PLK1 is a member of the polo-like kinase (PLK) family of serine/threonine kinases and is a key regulator of the cell cycle. PLK1 is intimately involved in numerous steps of mitosis, in the DNA damage response, and in DNA replication [8, 9]. Furthermore, PLK1 has been shown to be over expressed in numerous cancers, including AML [10]. In AML patients, PLK1 inhibition induces dose-dependent G2-M arrest and subsequent cell death via apoptosis [10]. Early pan-PLK1 inhibitors with activity against PLK2 and PLK3, although promising in preclinical studies, had high toxicity profiles and failed in the clinic [11].

Onvansertib (ONV) is a next-generation, highly selective ATP-competitive PLK1 inhibitor that has shown activity in AML cell lines and AML xenografts [12, 13]. The short in vivo half-life of onvansertib (~24 h) and oral bioavailability allows flexible dosing schedules and hence the potential to optimize the therapeutic window whilst minimizing toxicities.

A multicenter phase 1b/2 study (NCT03303339) was established to assess the safety, pharmacokinetics, and clinical activity of ONV in combination with either DAC or LDAC in patients with R/R AML. The phase 1b aspect of that study has previously been reported [14], and we refer the reader to that study for details. In phase 2, the safety and efficacy of the combination of ONV (60 mg/m^2^) and DAC was explored.

## Objectives

Here, we focus on the use of correlative studies from patients across the phase 1b/2 trial that received ONV + DAC, to identify molecular predictors of response to ONV + DAC. Using RNA-Seq data derived from blood samples collected at baseline, we developed a gene expression signature predictive of patient response to ONV + DAC. We then applied this signature to independent patient gene expression datasets: (1) to identify the mutational status of AML-associated genes in those patients predicted to respond and (2) to verify that the gene signature predicts response to the ONV + DAC combination, and not merely response to DAC as a single agent. We then verified the predicted AML mutational spectrum in the phase 1b/2 trial patients using the results of targeted sequencing.

## Materials and methods

**Fig. 1:**
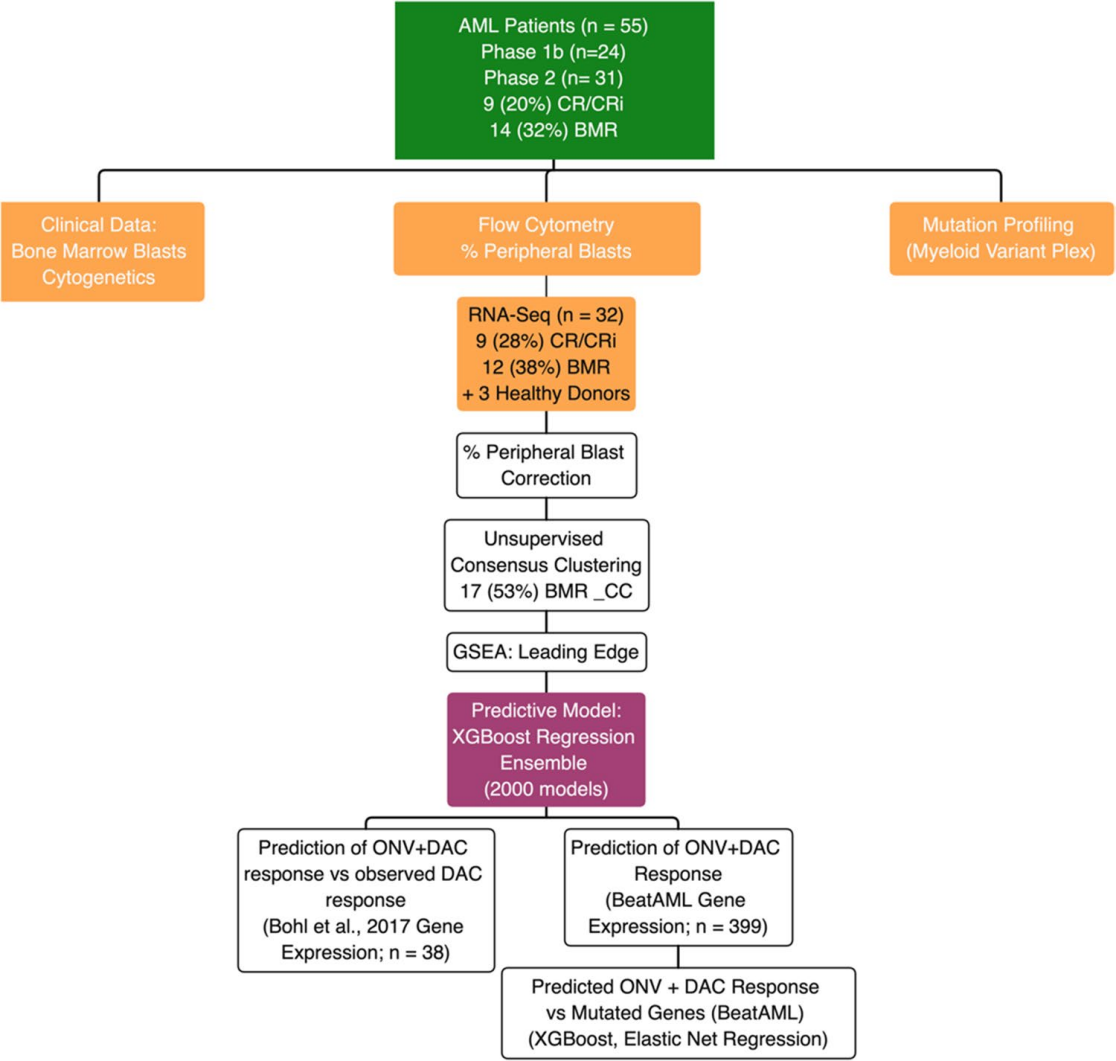
Analytical process outline

For an analytical outline see Fig. 1.

### Patient eligibility and treatment

For patient eligibility criteria, please refer to the phase 1b trial [14]. We note that up to three prior treatments for AML disease were permitted in phase 1b, and up to one prior treatment in phase 2, including HMA and VEN. Patients who were treatment naïve and not candidates for intensive induction therapy were also eligible. Onvansertib was administered orally on days 1 through 5. DAC was administered at 20 mg/m^2^ intravenously over 1 h, also on days 1 through 5. The treatment cycle was 28 days but could be shortened to 21 days if the investigators deemed that more frequent dosing could benefit the patient. Onvansertib doses ranged from 12 to 90 mg/m^2^ according to the phase 1b dose escalation with all phase 2 patients receiving the recommended phase 2 dose of 60 mg/m^2^ (Supplemental Table S1). Safety and efficacy assessments were also as in the phase 1b trial [14]*.* Patients were considered evaluable for efficacy if they had successfully completed at least one cycle of treatment.

### Bone marrow evaluation and anti-leukemic activity

Bone marrow aspirates were taken at screening, between 15 and 28 days of cycles 1 and 2, and following every other subsequent cycle, if considered appropriate by the investigator. Response to treatment was evaluated by the investigators using the modified International Working Group criteria [15] (see [14]). For biomarker analysis, we additionally defined “bone marrow response” (BMR) as ≥50% drop in bone marrow blast counts from baseline screening whilst on study.

### Blood collection and processing

Blood samples were collected from patients on day 1 (prior to treatment) of the first treatment cycle and processed 24 h after collection at Cardiff Oncology. For AML blast cell enumeration, blood samples were collected in CellSave Preservative Tubes (Silicon Biosystems); for genomic DNA extraction, samples were collected in EDTA tubes; and, for RNASeq, samples were collected into PAXgene tubes (Qiagen). AML blast percentages were quantified by fluorescence-activated cell sorting (FACS) using both a low side scatter/CD45^dim^ profile and the expression of blast markers as previously described [14].

Genomic DNA (gDNA) was extracted from peripheral blood mononuclear cells (PBMCs) and bone marrow mononuclear cells (BMMCs) and subject to targeted sequencing of 75 AML-associated genes using the Archer Myeloid VariantPlex system, also as previously described [14].

For RNA-Seq, total intracellular RNA was extracted from 32 of the 55 patients (Supplemental Table S1), plus from PBMCs from three healthy donors (HD), using the PAXgene Blood RNA Kit (Qiagen). Following pre-treatment with ezDNase (ThermoFisher), cDNA synthesis was carried out using the SMART-Seq v.4 Ultra Low Input RNA Kit (Takara Bio). Next-generation sequencing libraries were generated using the Nextera XT DNA Library Preparation kit (Illumina) and sequenced (paired-end 100bp) on a HiSeq4000 (Illumina). RNASeq data is available under the following GEO accession number: GSE239678.

### Gene expression and peripheral blast correction

Transcript expression quantification was estimated by quasi-mapping of raw reads to the human reference transcriptome GRCh38 (GENCODE v.36) [16] using Salmon (v1.4.0) [17] and converted to gene counts (length scaled transcripts per million, TPM) using tximport [18]. Gene counts were normalized for library size and, to limit the effect of skewness and mean-variance dependency, were variance-stabilizing transformed (VST) [19] using DESeq2 [20] in R.

Peripheral blood samples from AML patients contain a variable proportion of leukemic blast cells. Principal component analysis (PCA) was therefore used to control for this variation in the gene expression data by removing the principal components (PCs) that were most strongly correlated with %peripheral blasts (%PB). The variation explained (coefficient of determination, *R*^2^) between the %PB (including HD with 0%PB) and each PC was used as a weighting to, in turn, adjust the percent of overall variation in the expression data that was explained by each PC (*POV*), i.e., *R*^*2*^ × *POV*. The PCs were then sorted by their weighted *POV*, and the cumulative sum calculated. Those PCs most strongly associated with %PB (those with the largest weighted *POV*) were identified by the cumulative sum inflection point [21] and the data were then back-transformed excluding these PCs.

Disease manifestation may result from sample-specific modulation of different genes within a particular biological pathway; this potential noise was accounted for by converting the gene counts to pathway enrichment scores, using gene set variation analyses (GSVA v1.38) [22]. GSVA used 21,693 gene sets from the Molecular Signatures Database (MSigDB v.7.1): hallmarks (H), canonical pathways (C2), regulatory target gene sets (C3), cancer gene neighborhoods (C4), GO gene ontology (C5), oncogenic signatures (C6), and immunologic signatures (C7). The 1345 most variable, %PB corrected, GSVA scores were then used as input for unsupervised (*k* = 2) consensus clustering (“ConsensusClusterPlus” in R; Supplemental Fig. S1).

### Gene set enrichment analyses and gene signature modeling

The %PB corrected gene expression VST count data and the clustered BMR (BMR_CC vs No-BMR_CC; see “Results” and Supplemental Fig. S1) sample categorization were analyzed for differential gene expression using *limma’s* [23, 24] moderated *t* tests and results ranked by the signed significance score (log_2_ Fold Change × −log_10_(*P* value)) [25]. The ranked data was used as input for a gene set enrichment analysis (GSEA) [26, 27] against the MSigDB v.7.1 gene set collections. Gene sets with an adjusted *P* value [28] < 0.05 were retained, and the 3433 leading-edge genes that contributed to the peak enrichment score were collated. The leading-edge genes were then filtered to exclude genes not present in the BeatAML RNA-Seq data [29] (see below), ranked by their absolute significance score, and the inflection point used as a cut-off to yield 266 genes.

**Fig. 2:**
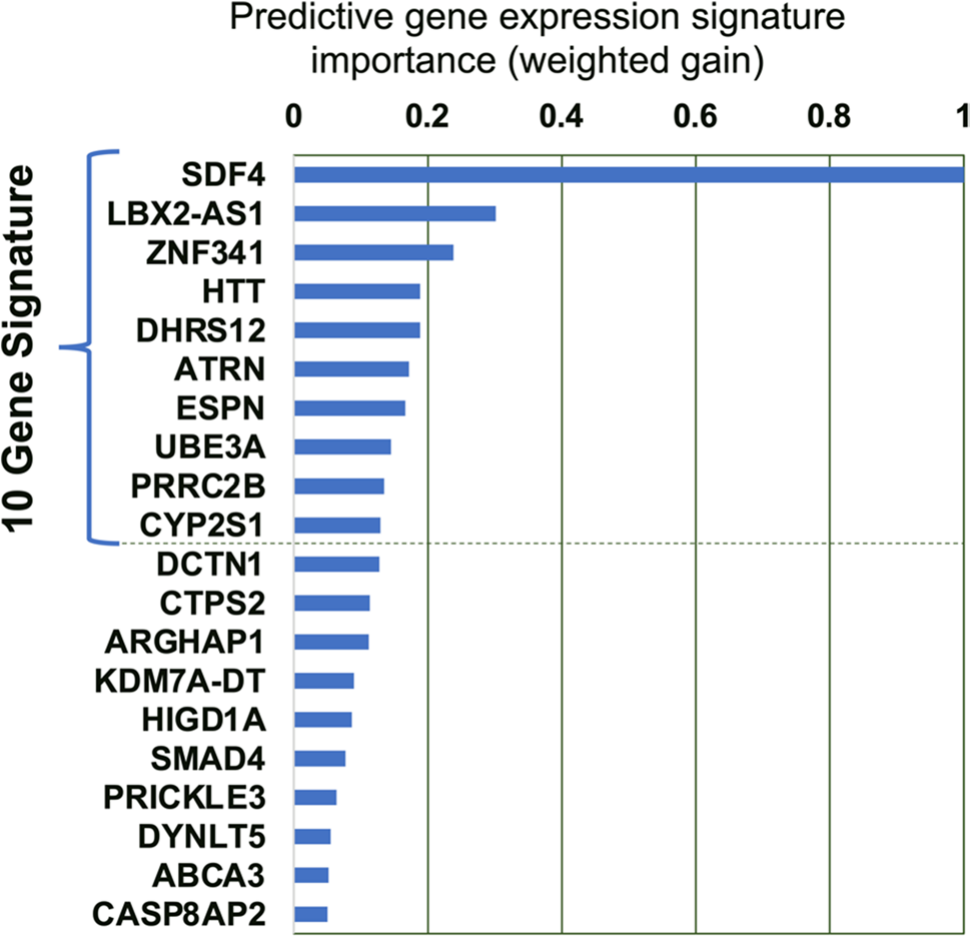
Variable (gene) importance from XGBoost. Variable importance shown for the top 20 genes (mean weighted gain from 2000 iterations (nested cross-validation) from the second round of feature selection). The top 10 genes used as the final gene signature are indicated

Model selection utilized gradient-boosted decision trees (XGBoost 1.5.2 [30]) for regression-based classification. Prior to machine learning, the count data were Blom (inverse normal) transformed. As the sample size was small (*n* = 32), a nested validation scheme was used (see Supplemental Fig. S2). In brief, the samples were *a* priori divided into an inner train set (80%) and an outer test hold-out set (20%). This was done randomly and repeated 100 times to yield 100 train-test data set pairs that were retained throughout the analysis. Genes (“features”) were selected and ordered via two rounds of feature selection on the inner train sets. In the first round of feature selection, each inner set was randomly divided into a training (80%) and a validation (20%) set and XGBoost run with default hyperparameters. This process was repeated 20 times and genes ordered by their mean gain across all 2000 iterations (20 × 100). Next, the top 50 genes were selected and the XGBoost hyperparameters optimized using repeated cross-validation (5 folds × 4 = 20). The top 50 genes were subject to a second round of feature selection analogous to the first except that the tuned hyperparameters were used and the top 10 genes extracted (Fig. 2). The hyperparameters for the 10 genes were then also optimized. Final models were generated using both the top 10 and top 50 selected genes using repeated cross-validation (5 folds × 4 = 20) on each of the 100 inner sample sets. Performance metrics were gathered with each cross-validation test set, and for predictions on the corresponding 100 hold-out test sets. These indicated that the 10-gene models were sufficient and performed better than the 50-gene models. The final model (“the gene signature”) was the ensemble of these 2000, 10-gene, models (Supplemental Fig. S2). Each predicted sample was characterized as a responder or non-responder based on the mode of the probability density distribution for that sample across all 2000 models.

### Predicting response to onvansertib plus decitabine in the BeatAML cohort

In order to explore the utility of the gene expression signature in an external dataset, it was applied to publicly available RNASeq data from 399 primary AML patient specimens in the BeatAML [29] project. The count data (CPM) were Blom transformed before applying the gene signature. If the gene signature is truly predictive, then it might be expected to correlate with the mutational status of genes in the samples. Genes that were mutated at least 3 times across the samples were selected (179 of 3333 genes), and dichotomously coded (non-mutated vs mutated). The mutated genes most strongly associated with the gene signature were identified by two approaches. First, regularized regression (elastic net) using glmnet (v4.1) and caret (v6.0) in R was used to identify 20 genes. Second, XGBoost was used to identify 17 genes. Being a tree-based algorithm, XGBoost can detect more complex interactions (e.g., epistasis) among genes than linear regression.

## Results

### Study population

A total of 55 patients were treated with onvansertib in combination with decitabine (ONV + DAC): 24 in phase 1b and 31 in phase 2. Baseline characteristics are summarized in Table 1 and in more detail in Supplemental Table S1. The overall median age of patients was 71 years (range, 23–85) and 34 (62%) were male. 32 (58%) of patients had an adverse risk cytogenetic profile at enrollment based on 2017 ELN recommendations [31]. Overall, 6 (11%) of patients had untreated AML, 34 (62%) had received one prior regimen, and 15 (27%) had received two or more treatments. The proportion of patients with ECOG 2 was higher in phase 2 patients (31%) than in phase 1b patients (4%). Phase 2 patients had also more often received HMA (DAC or AZA) treatment and/or VEN prior to study entry. Overall, 17 (31%) patients had received HMA and 8 (15%) had received VEN; all patients receiving VEN had also received HMA. The overall median percentage of bone marrow myeloblasts was 32% (range, 3–95%) and the median percentage of circulating peripheral blasts was 19.4 (range, 1.3–95.0%) (Supplemental Table S1).

**Table 1:** Patient enrollment and baseline characteristics

N (%) or median (range)	Phase 1b (*n* = 24)	Phase 2 (*n* = 31)	Phase 1b/2 combined (*n* = 55)
Age, years	66 (33–81)	73 (23–85)	71 (23–85)
Male gender	15 (65%)	19 (59%)	34 (62%)
ECOG 0–1	22 (96%)	22 (69%)	44 (80%)
ECOG 2	1 (4%)	10 (31%)	11 (20%)
Prior treatment			
0	4 (17%)	2 (6%)	6 (11%)
1	6 (25%)	28 (90%)	34 (62%)
2+	14 (58%)	1(3%)	15 (27%)
Prior HMA treatment (AML/MDS)	3 (13%)	14 (45%)	17 (31%)
Prior venetoclax treatment (AML)	0 (0)	8 (26%)	8 (15%)
Cytogenetic risk			
Favorable	1 (4%)	4 (13%)	5 (9%)
Intermediate	8 (35%)	9 (28%)	17 (31%)
Adverse	14 (61%)	18 (56%)	32 (58%)
Unknown	0 (0%)	1 (3%)	1 (2%)

For patient details see Supplemental Table S1

### Clinical responses and consensus clustering

Clinical responses for each of the 55 patients are summarized in Table 2 (see Supplemental Table S1). Of 44 evaluable patients, 9 (20%) patients achieved complete remission, with or without complete hematopoietic recovery (CR/CRi). Seven patients had a CR and two patients a CRi. The overall response rate (ORR), including CR, CRi, morphologic leukemia-free state (MLFS), and partial response (PR), was 27% (12/44 patients). Fourteen (32%) of the 44 evaluable patients exhibited a ≥50% reduction in bone marrow myeloblasts, defined as bone marrow response (BMR).

**Table 2:** Clinical responses to ONV + DAC by AML patients

Response*	All evaluable patients (*n* = 44)	RNA-Seq cohort (*n* = 32)*
CR/CRi	9 (20%)	9 (28%)
ORR	12 (27%)	12 (38%)
BMR	14 (32%)	13 (41%)
BMR_CC	NA	17 (53%)

*Note, the RNASeq subset was selected to enrich BMR and therefore does not reflect actual population response rates. *ORR includes CR, CRi, MLFS, PR. *BMR*, bone marrow response (≥50% decrease in blasts); *BMR_CC*, consensus clustered bone marrow response

Thirteen evaluable patients had received prior HMA therapy. No patients with a CR or CRi had previously been treated with an HMA (AZA, or DAC; *P* = 0.041; Table 3), and prior HMA treatment was equally counter indicative in terms of bone marrow response with zero BMR having prior HMA exposure (*P* = 0.003; Table 3). Seven evaluable patients had received prior VEN therapy and, as expected, all of these had also received prior HMA. No VEN-treated patient exhibited a response to ONV + DAC, though sample sizes were too small to achieve significance (Table 3).

**Table 3:** Stratified clinical responses to ONV + DAC

A. Clinical responses to ONV + DAC in relation to prior HMA exposure					
Response*	Prior HMA exposure (*n* = 13)	No prior HMA exposure (*n* = 31)	Total (*n* = 44)	Odds ratio (95% CI)	*P* value
CR/CRi	0 (0%)	9 (29%)	9 (20%)	0 (0–1.05)	0.041
ORR	1 (7%)	11 (35%)	13 (30%)	0.30 (0–1.34)	0.075
BMR	0 (0%)	14 (45%)	14 (32%)	0 (0–0.50)	0.003
B. Clinical responses to ONV + DAC in relation to prior venetoclax exposure					
Response	Prior VEN exposure (*n* = 7)	No prior VEN exposure (*n* = 37)	Total (*n* = 44)	Odds ratio (95% CI)	*P* value
CR/CRi	0 (0%)	9 (24%)	9 (20%)	0 (0–2.68)	0.314
ORR	0 (0%)	12 (32%)	12 (27%)	0 (0–1.74)	0.163
BMR	0 (0%)	14 (61%)	14 (32%)	0 (0–1.36)	0.078
C. Clinical responses to ONV + DAC in relation to splice factor mutations					
Response	SRSF2 or SF3B1 mutations (*n* = 12)	Other mutations (*n* = 32)	Total (*n* = 44)	Odds ratio (95% CI)	*P* value
CR/CRi	6 (50%)	3 (9%)	9 (20%)	9.00 (1.45–72.38)	0.007
ORR	8 (67%)	4 (13%)	12 (27%)	12.82 (2.27–92.86)	0.001
BMR	7 (58%)	7 (22%)	14 (32%)	4.79 (0.97–26.27)	0.032

*ORR includes CR, CRi, MLFS, PR. *BMR*, bone marrow response (≥50% decrease in blasts)

Baseline blood samples from 32 patients with sufficient RNA were analyzed by RNA-Seq, including 9 patients with CR/CRi and 13 with bone marrow response (BMR) (Table 2). To account for the variability in peripheral blasts between patients (Supplemental Table S1), the gene expression data were corrected based on the % of peripheral blasts at baseline (refer to Methods for details). Consensus clustering, a form of unsupervised class discovery, permits the discovery of natural data-driven groupings without supervision and a priori defined phenotypic classes. Since it is possible that some individuals exhibiting no BMR might have the genetic characteristics of responders and may have responded had other factors been different (e.g., their physical condition when entering the trial, their dosing scheme), consensus clustering was used to (1) verify the existence of 2 (*k*) clusters corresponding to bone marrow response (BMR_CC) and no bone marrow response (no-BMR_CC) and (2) reassign patients’ responses. This process resulted in 3 no-BMR being reassigned to BMR_CC, 2 non-evaluable (NE) to BMR_CC, and 1 NE to no-BMR_CC giving 17 (53%) BMR_CC and 15 (47%) no BMR_CC (Table 2 and Supplemental Table S1).

### Gene expression signature associated with sensitivity to ONV + DAC

Gene Set Enrichment Analysis (GSEA) was carried out (1) to gain insight into key pathways that are differentially regulated between ONV + DAC responders (BMR_CC) and non-responders and (2) to identify leading-edge genes as the basis for predictive model building (see “Methods”). Responders to the ONV + DAC combination were enriched for oxidative phosphorylation (OXPHOS), mitochondrial function, and protein synthesis (Supplemental Figs. S3–S6).

The RNAseq data from the 32 patients was next used to generate a 10 gene signature (SDF4, LBX-AS1, ZNF341, HTT, DHRS12, ATRN, ESPN, UBE3A, PRRC2B, and CYP2S1) predictive of ONV + DAC response (Fig. 2, Supplemental Fig. S2). The 10-gene signature was realized as an ensemble of 2000 XGBoost models.

In order to confirm that the gene expression signature of response to ONV + DAC was likely indicative of response to the combination and not merely predictive of response to DAC alone, the ONV + DAC gene signature was applied to Blom transformed array data (GEO GSE84334) from a study from Bohl et al. [32] in which AML patients were subject to single agent DAC therapy in a first line setting. The predicted response to ONV + DAC was contrasted with the known response to DAC in the 38 evaluable AML patients from the Bohl data (Supplemental Tables S2–S3). Eighteen patients in the Bohl study had shown a response to DAC (CR = 4, PR = 6, blast reduction > 25% = 8), and 20 patients had no response. There was no association between the observed response to DAC and the predicted response to ONV + DAC (*P* = 0.7449; see Supplemental Table S3)—indicating that the gene signature is not predictive of response to DAC single agent and is likely to reflect sensitivity to the combination of ONV + DAC.

### Gene expression signature is associated with spliceosome mutations in an independent AML cohort

**Fig. 3:**
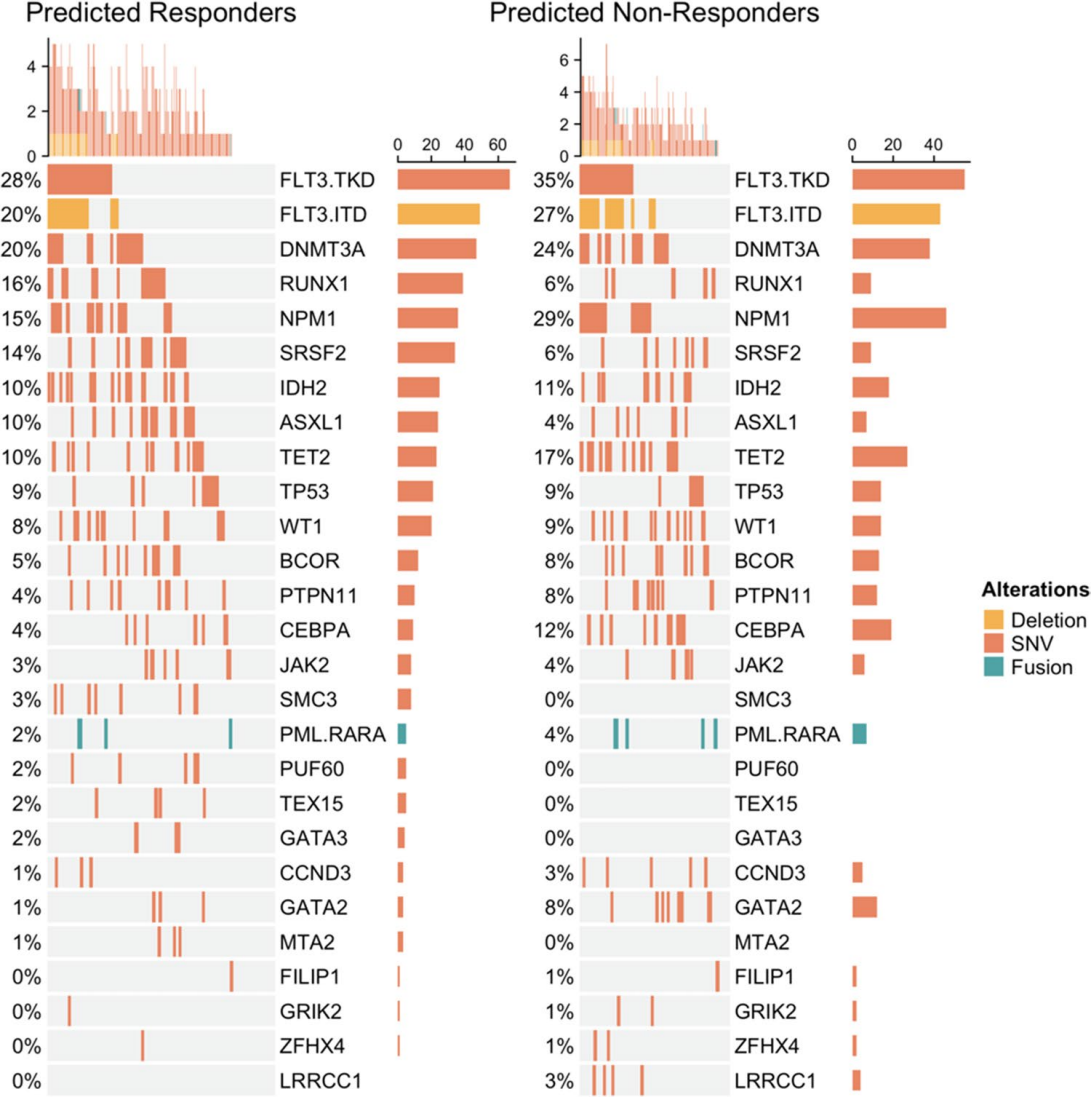
Heatmap showing the prevalence of 27 mutated genes in the BeatAML cohort that were associated with the ONV + DAC predictive gene signature

The gene signature model ensemble was then applied to gene expression data from 399 patients in the BeatAML cohort [29]. It predicted 241 putative responders to ONV + DAC and 158 putative non-responders. For each BeatAML sample, its predicted probability of responding to ONV + DAC was then regressed against the set of 179 AML genes that were mutated at least 3 times across the samples (see “Methods”) resulting in a set of 27 associated, mutated genes. A summary multivariate linear regression summarizes the effects of these 27 genes (Table 4), and their incidence in the BeatAML cohort is illustrated in Fig. 3.

**Table 4:** Multiple linear regression of 27 mutated AML genes associated with gene expression–predicted ONV+DAC response in BeatAML (*n* = 399)

Mutated gene	Coefficient	*P* value
SRSF2	0.0929	**0.0009**
GATA3	0.1383	0.0764
PUF60	0.1185	0.0900
SMC3	0.0952	0.0902
MTA2	0.1116	0.2231
TEX15	0.0758	0.2849
RUNX1	0.0395	0.1363
ASXL1	0.0248	0.4175
DNMT3A	0.0070	0.7319
WT1	0.0028	0.9192
FLT3 (ITD)	−0.0002	0.9928
TP53	−0.0221	0.4318
JAK2	−0.0327	0.4467
FLT3 (TKD)	−0.0274	0.2355
IDH2	−0.0318	0.2217
PTPN11	−0.0551	0.1202
TET2	−0.0496	**0.0399**
BCOR	−0.0643	0.0587
CEBPA	−0.0694	**0.0304**
LRRCC1	−0.1272	0.0997
CCND3	−0.1036	0.0579
GATA2	−0.0914	**0.0302**
NPM1	−0.0636	**0.0042**
FILIP1	−0.1770	**0.0426**
PML-RARA	−0.1265	**0.0057**
GRIK2	−0.1958	**0.0306**
ZFHX4	−0.2687	**0.0023**

Mutated genes associated with predicted response were selected by regularized regression (elastic net) and a simple XGBoost regression model and then summarized by simple multivariate linear regression as shown here. Bold = *P* value < 0.05

One gene, the splicing factor SRSF2, was significantly and positively associated with the predicted response to ONV + DAC (*P* = 0.0009). Other mutated genes positively related to the gene signature included GATA3 which regulates the balance between self-renewal and differentiation in hematopoietic stem cells [33], the splicing factor PUF60, and the cohesion factor SMC3. This suggested that spliceosome factors could be important in the response to ONV + DAC. The prevalence of the spliceosome genes SRSF2, and the less frequently mutated SF3B1, U2AF1, and ZRSR2, was therefore examined in the BeatAML samples. Twenty-two percent (86 of 399) of BeatAML samples carried a spliceosome mutation and 73% (63) of these were predicted to be responders to ONV + DAC, confirming that splicing factor genes, as a group, were associated with the predicted response in the BeatAML cohort (*χ*^2^_df=1_ = 4.46, *P* = 0.035).

### SRSF2 and SF3B1 mutations are associated with response to ONV + DAC in AML

The mutational profiling of the ONV + DAC cohort was performed at baseline for all patients (*n* = 55) using DNA from PBMCs or BMMCs. The most frequently mutated genes were ASXL1 (22%), SRSF2 (22%), TP53 (16%), NRAS (16%), FLT3_ITD (15%), FLT3_TKD (13%), TET2 (11%), and DNMT3A (11%; see Supplemental Tables S4 and S5).

Given the putative association observed between the predicted probability of ONV + DAC response and splice factor genes in BeatAML, the mutational status of the core splice factor genes SRSF2 and SF3B1 was examined in the ONV + DAC cohort. Indeed, patients with SRSF2 or SF3B1 mutations were 9.0 times more likely to be responders (CR/CRi) than not (*P* = 0.007; Table 3). When the overall response rate (CR/CRi/MFLS/PR) was considered, patients carrying a SRSF2 or SF3B1 mutation were 12.8 times more likely to be responders than not (*P* = 0.001; Table 3). Mutations in the splice factor gene ZRSR2 were not observed in the phase 1b/2 study, and the splice factor U2AF1 was only mutated in 3 non-responders.

## Discussion

In this study, of R/R AML patients enrolled in a phase 1b/2 clinical trial for combination treatment with onvansertib plus decitabine (ONV + DAC), we derived gene expression data using bulk RNAseq from circulating, peripheral myeloid blasts. After correcting the data for the varying percentage of blasts present, transforming the gene counts, and subjecting the expression data to unsupervised clustering, we used nested, tree-based boosted regression (XGBoost) to build an ensemble of 2000 models that could be used to predict response to ONV + DAC.

The initial set of genes (features) for model building was selected using the leading edge from GSEA on multiple gene sets. Given limited and somewhat heterogenous samples, GSEA provides a robust way to identify gene sets/pathways that are enriched in association with a response, and the leading edge identified those genes contributing to that enrichment. The final set of 10 genes was selected through two rounds of model building and variable selection. The final 2000 model ensemble had 100% recall on the full original (consensus clustered) dataset, reflecting the value of a model ensemble. However, despite attempts to minimize over-fitting through the judicious use of a nested and cross-validated design, we acknowledge that having only 32 samples necessitates the re-using of samples for training and testing making some model over-fitting inevitable.

In addition to providing the initial set of genes for predictive modeling building, GSEA also indicated that ONV + DAC responders had elevated mitochondrial function, most notably oxidative phosphorylation (OXPHOS). OXPHOS addiction in AML cells is known to be associated with chemotherapy resistance and adverse prognosis in AML. OXPHOS-addicted AML cells are targeted by VEN + HMA through inhibition of electron transport chain complex II [34]. High OXPHOS AML cells are also typically glucose addicted through ATP inhibition of AMPK and activation of mTORC1 [35, 36], and they use the pentose phosphate pathway (PPP) to metabolize glucose. Since PLK1 is known to phosphorylate and activate G6PD [37], this represents a possible way in which PLK inhibition could target high OXPHOS AML cells, and warrants further investigation.

When the ONV + DAC gene signature was applied to the external BeatAML cohort, predicted responders were characterized by enrichment of mutations in the splice factor gene *SRSF2* (Table 4; Fig. 3). Mutations in other core splicing factor genes (*SF3B1*, *U2AF1*, *ZRSR2*) were also somewhat enriched in the predicted responders. In the ONV + DAC–treated patients examined in the current study, we observed that patients with SRSF2 or SF3B1 mutations were 9 times more likely to be responders (CR/CRi) than not (*P* = 0.007; Table 3), with that value increasing to 12.8 times (*P* = 0.001) when overall response rate (CR/CRi/MLFS/PR) was considered.

Spliceosome mutations (such as *SRSF2*, *SF3B1*, *U2AF1*, and *ZRSR2*), along with driver mutations in the cohesion complex, transcriptions factors (*RUNX1*), and chromatin modifiers (e.g., *ASXL1*), define a chromatin-spliceosome molecular subtype of AML (CS-AML). Overlapping characteristics are also frequently observed in MDS and secondary AML [38, 39]. Spliceosome mutations define a high-risk AML subtype that tends to have poor outcomes to intensive chemotherapy, and are found more frequently in older patients for whom intensive treatment may not be an option [2, 39, 40]. However, the presence of spliceosome mutations in the HMA + VEN setting has been shown to provide outcomes for patients with spliceosome mutations that are at least as favorable as wildtype (ORR of 89% vs 79%)^2^. SRSF2 and SF3B1 mutations alter the normal sequence-specific RNA binding activity of their proteins driving aberrant splicing [41]. Aberrant splicing (AS) has also been shown to be linked to both cell cycle control and apoptosis, suggesting direct relationships between AS and cell cycle control agents including PLK1 [42].

In the current study, no patient who had received prior HMA, or HMA + VEN, responded to ONV + DAC. This would suggest that these patients were either resistant or had become resistant to HMA or HMA + VEN, and that the failure of these patients to respond to ONV + DAC results from this prior HMA resistance. Also, although HMA + VEN has been shown to be efficacious following HMA failure, ONV + DAC would appear unlikely to be able to rescue HMA + VEN failure.

Given this, one may ask if the observed ONV + DAC responders were largely responding to DAC and not specifically to the combination. Although we cannot know whether the (HMA naïve) patients in the current study would, or would not, have responded to DAC as a single agent, several lines of evidence suggest that it is highly unlikely that the ONV + DAC responders were simply responding to DAC and not to the combination. First, when the response to ONV + DAC was predicted in patients with a known status of response to single agent DAC [32], neither predicted ONV + DAC responders nor predicted non-responders were associated with known DAC response (Supplemental Tables S2 and S3). Second, additional support comes from the observation that none of the responders to ONV+DAC carried *TET2* or *TP53* mutations (Supplemental Table S5), although both *TET2* and *TP53* mutations have previously been associated with favorable response to HMA in AML and MDS [43, 44]. Chronic myelomonocytic leukemia (CMML) is a hybrid myeloproliferative/myelodysplastic with a tendency to progress to AML [45]. Like MDS and cs-AML, it is characterized, in up to 80% of cases, by mutations both in epigenetic modifiers, notably ASXL1 truncations leading to highly proliferative phenotypes, and SF genes [45, 46]. Consequently, safety and preliminary efficacy of onvansertib in R/R CMML are being studied in a phase 1 trial [NCT05549661].

## Conclusion

Our results suggest that ONV + DAC has preliminary efficacy as a treatment for R/R AML in patients who have not received prior HMA (DAC or AZA, either alone or in combination with VEN). The efficacy of ONV + DAC is not predicted by the efficacy of DAC as a single agent; however, prior HMA failure may predict lack of response to ONV + DAC. A gene expression signature–based model, applied to an independent dataset predicted that mutations in splice factor genes, notably SRSF2 and SF3B1, may be predictive of ONV + DAC response. This association was verified in ONV + DAC–treated patients and warrants further investigation.

### Supplementary information

ESM 1 (PDF 1166 kb)

## Data Availability

The data generated during the current study were deposited into the Gene Expression Omnibus (GEO) database under accession number GSE239678 and are available at the following URL: https://www.ncbi.nlm.nih.gov/geo/query/acc.cgi?acc=GSE239678. The data from Bohl et al. [32] was taken from GEO GSE84334 at the following URL: https://www.ncbi.nlm.nih.gov/geo/query/acc.cgi?acc=GSE84334. The beatAML gene expression data and mutation data were taken from that publication’s supplemental information tables: 10.1038/s41586-018-0623-z. All other data are included in this published article [and its supplementary information files]. Further information will be made available upon reasonable request to the corresponding author.
