# Supplementary material for: Spliceosome mutations are associated with clinical response in a phase 1b/2 study of the PLK1 inhibitor onvansertib in combination with decitabine in relapsed or refractory acute myeloid leukemia
Source: Ann Hematol. 2023 Sep 13;102(11):3049–59. doi: 10.1007/s00277-023-05442-9 (PMC10567832; doi:10.1007/s00277-023-05442-9)
Supplement: Supplementary file 1 — (PDF 1166 kb) [file 277_2023_5442_MOESM1_ESM.pdf]

Supplementary Information for:

Spliceosome mutations are associated with clinical response in a Phase 1b/2 study of the PLK1 inhibitor onvansertib in combination with decitabine in relapsed or refractory acute myeloid leukemia.

Peter J P Croucher, DPhil[1]; Maya Ridinger, PhD[1]; Pamela S. Becker MD, PhD[2]; Tara L. Lin, MD[3]; Sandra L. Silberman, MD, PhD[4]; Eunice S. Wang, MD[5]; Amer M. Zeidan, MBBS, MHS[6]

Addresses:

[1]Cardiff Oncology Inc., 11055 Flintkote Avenue, San Diego, CA 92121

[2]Leukemia Division, Department of Hematology and Hematopoietic Cell Transplantation, City of Hope National Medical Center, Duarte, CA

[3]Division of Hematologic Malignancies and Cellular Therapeutics, University of Kansas, Kansas City, Kansas

[4]SLS Oncology LLC, Durham, North Carolina

[5]Leukemia Service, Roswell Park Comprehensive Cancer Center, Buffalo, NY.

[6]Amer Zeidan: Yale University and Yale Cancer Center, New Haven, Connecticut.

Corresponding Author: Amer M. Zeidan, Yale University, 333 Cedar Street, PO Box 208028, New Haven, CT 06520-8028. Phone: 203-737-7103; Fax: 203-785- 7232; E-mail: amer.zeidan@yale.edu

Table of Contents

Supplemental Tables 3

Supplemental Table S1 Baseline characteristics and responses for all patients 4

Supplemental Table S2 Contrast between predicted ONV + DAC response and observed DAC response in DAC treated AML patients 7

Supplemental Table S3 Contrast between predicted ONV + DAC response and observed DAC response in DAC treated AML patients – contingency table 8

Supplemental Table S4 Summary of patient myeloid gene mutation profiling 9

Supplemental Table S5 Mutation profile of patients showing a response 10

References 10

Supplemental Figures 11

Supplemental Fig. S1 Unsupervised consensus clustering (CC) 12

Supplemental Fig. S2 Generation of a 10 gene predictive gene expression signature 13

Supplemental Fig. S3 Gene Set Enrichment Analysis (GSEA), Hallmarks of Cancer 14

Supplemental Fig. S4 Gene Set Enrichment Analysis (GSEA), Gene Ontology Biological Processes 15

Supplemental Fig. S5 Gene Set Enrichment Analysis (GSEA), Gene Ontology Molecular Function 16

Supplemental Fig. S6 Gene Set Enrichment Analyses (GSEA), Gene Ontology Cellular Component 17

Supplemental Tables

Supplemental Table S1: Baseline characteristics and responses for all patients

Phase	Pt_ID	Age	Sex	ONV DOSE (mg/m^2)	ECOG	Prior AML	Prior AZA	Prior DAC	Prior HMA	Prior VEN	Cytogenetic risk (ELN2017)	% Bone marrow blasts	% Peripheral Blasts (C1D1)	Best Response[1]	BMR	BMR_CC[2]
1b	01-021	77	MALE	18	1	1	YES	NO	YES	NO	Intermediate	60	5.8	NONE	NO	**YES**
1b	03-241	69	FEMALE	60	1	3	NO	YES	YES	NO	Favorable	46	9	NE	NE	—
1b	03-051	68	MALE	60	1	1	NO	NO	NO	NO	Adverse	12	19.4	NONE	NO	NO
1b	03-060	66	FEMALE	90	0	3	NO	YES	YES	NO	Adverse	22	1.3	NONE	NO	NO
1b	05-030	68	FEMALE	27	1	3	NO	NO	NO	NO	Intermediate	20	43.5	CRi	YES	**YES**
1b	05-043	66	MALE	40	1	1	NO	NO	NO	NO	Intermediate	19	82.5	PR	NO	NO
1b	07-008	48	FEMALE	12	1	2	NO	NO	NO	NO	Adverse	76	58.9	NE	NE	**YES**
1b	07-009	75	MALE	12	1	1	NO	NO	NO	NO	Adverse	94	92	CR	YES	**YES**
1b	07-011	62	MALE	12	1	0	NO	NO	NO	NO	Adverse	NE	NE	NONE	NO	NO
1b	07-013	33	MALE	12	1	2	NO	NO	NO	NO	Intermediate	27	58.3	NE	NE	NO
1b	07-018	65	FEMALE	18	2	1	NO	NO	NO	NO	Adverse	7	6.5	NONE	NO	NO
1b	07-033	49	MALE	27	1	2	NO	NO	NO	NO	Adverse	66	20.6	NE	NE	**YES**
1b	07-035	81	MALE	40	1	1	NO	NO	NO	NO	Adverse	22	1.3	CR	YES	**YES**
1b	07-036	75	FEMALE	40	1	0	NO	NO	NO	NO	Adverse	40	5.6	NONE	YES	**YES**
1b	07-038	63	MALE	40	1	2	NO	NO	NO	NO	Adverse	83	14.8	NE	NE	—
1b	07-062	70	MALE	90	0	3	NO	NO	NO	NO	Intermediate	20	2	NONE	NO	NO
1b	07-063	58	MALE	90	1	3	NO	NO	NO	NO	Adverse	22	13.2	NONE	NO	—
1b	08-050	64	MALE	60	1	3	NO	NO	NO	NO	Intermediate	82	78.8	NONE	NO	NO
1b	08-058	51	FEMALE	90	1	2	NO	NO	NO	NO	Adverse	11	3.8	CR	YES	**YES**

Continued…

Supplemental Table S1: Baseline characteristics and responses for all patients – *continued*

Phase	Pt_ID	Age	Sex	ONV Dose (mg/m^2)	ECOG	Prior AML	Prior AZA	Prior DAC	Prior HMA	Prior VEN	Cytogenetic risk (ELN2017)	% Bone marrow blasts	% Peripheral Blasts (C1D1)	Best Response[1]	BMR	BMR_CC[2]
1b	08-061	67	FEMALE	90	1	2	NO	NO	NO	NO	Favorable	NE	42.1	MLFS	YES	**YES**
1b	09-026	64	MALE	18	1	2	NO	NO	NO	NO	Adverse	20	54.6	NONE	NO	NO
1b	09-034	76	MALE	27	1	0	NO	NO	NO	NO	Intermediate	49	83.3	NONE	NO	NO
1b	09-064	76	MALE	90	1	0	NO	NO	NO	NO	Intermediate	10	1.9	CR	YES	**YES**
1b	12-048	60	FEMALE	60	1	2	NO	NO	NO	NO	Adverse	27	76.3	NONE	NO	NO
2	01-213	85	MALE	60	1	1	YES	NO	YES	NO	Adverse	77	18	NE	NE	—
2	01-235	74	MALE	60	1	1	YES	NO	YES	YES	Adverse	32	32	NONE	NO	—
2	03-219	71	MALE	60	2	1	NO	NO	NO	NO	Favorable	87	75	NONE	NO	NO
2	03-222	57	MALE	60	1	1	NO	NO	NO	NO	Favorable	NA	14	CRi	YES	**YES**
2	03-224	49	MALE	60	1	1	NO	NO	NO	NO	Adverse	NA	15	NONE	NO	—
2	03-238	57	FEMALE	60	1	1	NO	NO	NO	NO	Adverse	NA	2	CR	YES	**YES**
2	03-245	77	MALE	60	1	1	NO	YES	YES	NO	Adverse	71	70	NONE	NO	—
2	05-212	74	MALE	60	1	1	YES	NO	YES	NO	Adverse	34	56	PR	NO	NO
2	05-220	74	FEMALE	60	2	1	YES	NO	YES	NO	Intermediate	68	95	NE	NE	—
2	05-230	72	FEMALE	60	1	1	YES	NO	YES	YES	Adverse	10	16	NONE	NO	—
2	05-233	73	FEMALE	60	1	0	NO	NO	NO	NO	Intermediate	64	18	NONE	YES	—
2	05-239	77	MALE	60	2	0	NO	NO	NO	NO	Intermediate	64	29	NONE	YES	**YES**

Continued…

Supplemental Table S1: Baseline characteristics and responses for all patients – *continued*

Phase	Pt_ID	Age	Sex	ONV DOSE (mg/m^2)	ECOG	Prior AML	Prior AZA	Prior DAC	Prior HMA	Prior VEN	Cytogenetic risk (ELN2017)	% Bone marrow blasts	% Peripheral Blasts (C1D1)	Best Response[1]	BMR	BMR_CC[2]
2	05-243	75	FEMALE	60	2	1	YES	NO	YES	YES	Adverse	5	22	NONE	NO	—
2	07-214	70	FEMALE	60	1	1	NO	NO	NO	NO	NA	75	84	NONE	NO	NO
2	07-221	72	MALE	60	1	1	YES	NO	YES	YES	Adverse	15	4.3	NONE	NO	**YES**
2	07-242	68	FEMALE	60	1	1	YES	NO	YES	YES	Adverse	NE	65	NONE	NO	—
2	08-205	65	MALE	60	2	1	NO	NO	NO	NO	Adverse	70	2	CR	YES	**YES**
2	08-208	66	FEMALE	60	1	1	NO	NO	NO	NO	Adverse	45	2.1	NE	NE	—
2	08-211	23	FEMALE	60	2	1	NO	NO	NO	NO	Adverse	50	65	NE	NE	—
2	08-217	73	FEMALE	60	1	1	NO	NO	NO	NO	Adverse	9	2.6	CR	YES	**YES**
2	08-226	82	MALE	60	1	1	NO	NO	NO	NO	Intermediate	3	8	NONE	NO	—
2	09-202	70	MALE	60	2	1	NO	NO	NO	NO	Adverse	9	12.3	NONE	NO	—
2	09-227	73	MALE	60	2	1	NO	NO	NO	NO	Intermediate	95	84	NONE	YES	**YES**
2	09-228	77	FEMALE	60	0	1	NO	NO	NO	NO	Adverse	NE	NE	NONE	NO	—
2	11-225	72	MALE	60	1	2	NO	NO	NO	NO	Favorable	46	2	NONE	NO	—
2	12-203	77	MALE	60	0	1	NO	YES	YES	NO	Intermediate	55	83	NE	NE	—
2	12-204	74	FEMALE	60	1	1	YES	NO	YES	YES	Intermediate	7	25	NONE	NO	—
2	12-207	82	MALE	60	2	1	YES	NO	YES	YES	Adverse	13	36	NONE	NO	NO
2	12-209	80	MALE	60	1	1	NO	YES	YES	NO	Intermediate	32	7	NONE	NO	—
2	12-231	74	MALE	60	1	1	YES	NO	YES	NO	Adverse	25	17	NONE	NO	—
2	12-244	83	MALE	60	2	1	NO	YES	YES	YES	Adverse	30	79	NE	NE	—

Supplemental Table S1 notes: [1]CR = complete remission; CRi = complete remission without hematopoietic recovery; MLFS = morphologic leukemia free state; PR = partial response; NONE = no response; NE = non-evaluable. [2]Samples with an entry under BMR_CC were samples subject to RNASeq and consensus clustering to reclassify them into molecularly defined response groupings. These samples and classification formed the basis of the predictive modeling.

Supplementary Table S2. Contrast between predicted ONV + DAC response and observed DAC response in DAC treated AML patients (Bohl et al, 2017 [1])

GEO Accession	Age	Gender	Genetic_risk	DNMT3A	NPM1	Observed DAC Response	Predicted ONV + DAC Response
GSM2232030	74	M	INTERMEDIATE	WT	MUT	YES	NO
GSM2232031	75	F	HIGH	WT	-	YES	YES
GSM2232035	80	M	INTERMEDIATE	WT	MUT	YES	YES
GSM2232037	76	F	HIGH	-	-	YES	YES
GSM2232038	73	M	INTERMEDIATE	WT	WT	YES	YES
GSM2232039	85	M	HIGH	WT	WT	YES	YES
GSM2232041	69	F	INTERMEDIATE	-	WT	YES	YES
GSM2232045	76	F	HIGH	WT	WT	YES	YES
GSM2232046	72	M	INTERMEDIATE	WT	WT	YES	YES
GSM2232049	70	M	HIGH	WT	WT	YES	YES
GSM2232052	63	F	HIGH	WT	WT	YES	NO
GSM2232055	82	F	-	MUT	WT	YES	YES
GSM2232058	72	M	HIGH	WT	WT	YES	NO
GSM2232062	74	M	-	WT	WT	YES	YES
GSM2232063	61	M	HIGH	WT	WT	YES	NO
GSM2232068	69	F	INTERMEDIATE	MUT	WT	YES	NO
GSM2232069	66	M	-	WT	WT	YES	YES
GSM2232070	67	F	INTERMEDIATE	WT	WT	YES	NO
GSM2232029	65	M	INTERMEDIATE	WT	WT	NO	NO
GSM2232032	68	F	-	WT	WT	NO	YES
GSM2232033	70	M	-	WT	WT	NO	NO
GSM2232036	71	M	INTERMEDIATE	WT	WT	NO	YES
GSM2232040	66	M	HIGH	WT	WT	NO	YES
GSM2232043	69	M	INTERMEDIATE	WT	WT	NO	YES
GSM2232044	62	M	INTERMEDIATE	WT	MUT	NO	YES
GSM2232047	73	F	HIGH	WT	WT	NO	YES
GSM2232048	81	M	INTERMEDIATE	WT	WT	NO	YES
GSM2232051	71	M	-	MUT	WT	NO	YES
GSM2232053	65	F	INTERMEDIATE	WT	WT	NO	YES

Supplementary Table S2. Contrast between predicted ONV + DAC response and observed DAC response in DAC treated AML patients (Bohl et al, 2017 [1]) - *continued*

GEO Accession	Age	Gender	Genetic_risk	DNMT3A	NPM1	Observed DAC Response	Predicted ONV + DAC Response
GSM2232056	65	M	HIGH	-	WT	NO	NO
GSM2232059	80	F	HIGH	MUT	WT	NO	NO
GSM2232060	70	M	INTERMEDIATE	WT	MUT	NO	NO
GSM2232061	70	F	INTERMEDIATE	WT	WT	NO	NO
GSM2232064	80	M	INTERMEDIATE	WT	WT	NO	NO
GSM2232065	83	M	INTERMEDIATE	WT	MUT	NO	YES
GSM2232066	71	F	INTERMEDIATE	MUT	WT	NO	YES
GSM2232067	71	M	INTERMEDIATE	WT	WT	NO	NO
GSM2232071	74	M	-	MUT	WT	NO	YES

For genes with multiple mapping probe-sets, the probe-set with the maximum median absolute deviation was used, and the normalized RMA data were Blom transformed before applying the gene signature.

Supplementary Table S3. Contrast between predicted ONV + DAC response and observed DAC response in DAC treated AML patients (Bohl et al, 2017[1]) – contingency table:

	Predicted ONV + DAC Response	Predicted ONV + DAC Non-response
Observed DAC Response	12	6
Observed DAC Non-response	12	8

P-value (Fisher's exact test) = 0.7449

Supplementary Table S4. Summary of patient myeloid gene mutation profiling

Mutational Profile (n = 55)		
**Gene**	**Patients (n)**	**Patients (%)**
ASXL1	12	22
SRSF2	12	22
TP53	9	16
NRAS	9	16
FLT3_ITD	8	15
FLT3	7	13
TET2	6	11
DNMT3A	6	11
JAK2	5	9
NPM1	5	9
RUNX1	5	9
SF3B1	4	7
KRAS	4	7
NOTCH1	3	5
U2AF1	3	5
BCOR	3	5
SETBP1	2	4
KIT	2	4
NF1	2	4
GATA2	2	4
IDH2	2	4
WT1	2	4
CDKN2A	1	2
PTPN1	1	2
CBL	1	2
PHF6	1	2
CEBPA	1	2
PMS2	1	2
NTRK3	1	2
CFS3R	1	2
IDH1	1	2
SMC1A	1	2
DDX41	1	2

Supplemental Table S5. Mutation profile of patients showing a response

Patient_ID	Response Profile	Mutations
03-238	BMR_CC , BMR, CR	None
07-009	BMR_CC , BMR, CR	SF3B1, FLT3
07-035	BMR_CC , BMR, CR	SRSF2, ASXL1, IDH2
08-058	BMR_CC , BMR, CR	NRAS, FLT3_ITD
08-205	BMR_CC , BMR, CR	DNMT3A, FLT3_ITD
08-217	BMR_CC , BMR, CR	SRSF2, ASXL1
09-064	BMR_CC , BMR, CR	SF3B1
03-222	BMR_CC , BMR, CRi	None
05-030	BMR_CC , BMR, CRi	SRSF2, NRAS, CDKN2A
08-061	BMR_CC , BMR, MLFS	SF3B1, DNMT3A, FLT3_ITD, NPM1
05-239	BMR_CC, BMR, SD	SRSF2, NRAS,
07-036	BMR_CC, BMR, SD	TET2
09-227	BMR_CC, BMR, SD	FLT3, TET2
07-008	BMR_CC, NE, NE	ASXL1, FLT3
07-033	BMR_CC, NE, NE	TP53, KRAS
01-021	BMR_CC, No BMR, SD	ASXL1, TET2
07-221	BMR_CC , No BMR, SD	SRSF2, ASXL1, FLT3_ITD, SETBP1, NF1
05-043	No BMR_CC, No BMR, PR	SRSF2, NRAS
05-212	No BMR_CC, No BMR, PR	SRSF2, NRAS, FLT3_ITD, FLT3, TET2

BMR_CC = bone marrow response (consensus clustered); BMR = bone marrow response (≥ 50% reduction in myeloblasts); NE = not evaluated; SD = stable disease; all other categories refer to Table S1.

References

1. Bohl SR, Dolnik A, Jensen T, et al. Gene expression analysis of decitabine treated AML: high impact of tumor suppressor gene expression changes. *Leuk Lymphoma*. 2017;58(9):2264-2267. doi:10.1080/10428194.2017.1287360

Supplemental Figures

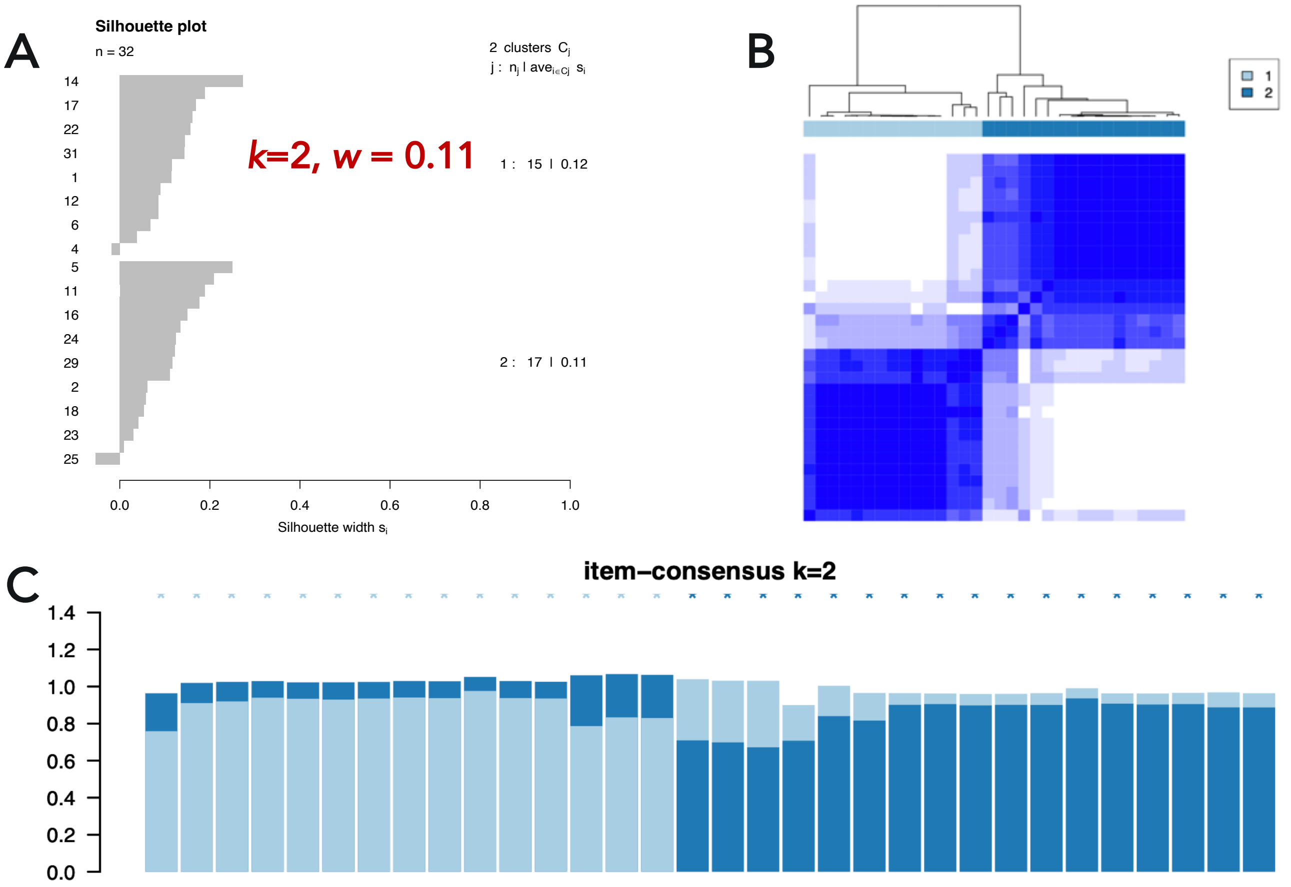

Supplemental Fig. 1 Unsupervised consensus clustering (CC) of 1345 most variable GSVA transformed, %PB corrected, gene counts confirmed that the data conformed to two clusters (*k*=2) corresponding to bone marrow responders (BMR_CC) and non-bone marrow responders (no-BMR_CC). Consensus clustering used the *k*-medoid algorithm (PAM), *k* = 2…10, 1000 iterations, sampling 80% of the variables and 80% of the samples at each iteration. A: Silhouette plot for *k* = 2 clusters. The silhouette score (w) for *k* = 2 was 0.11 and decreased for increasing values of *k* (*k* = 3, *w* = 0.06, *k* = 4, *w* = 0.07). B: Consensus matrix plot for *k* = 2 clusters. C: Item-consensus plot for each of the 32 RNA-Seq samples indicating each samples' proportion of identity with each of the *k* = 2 genetic clusters

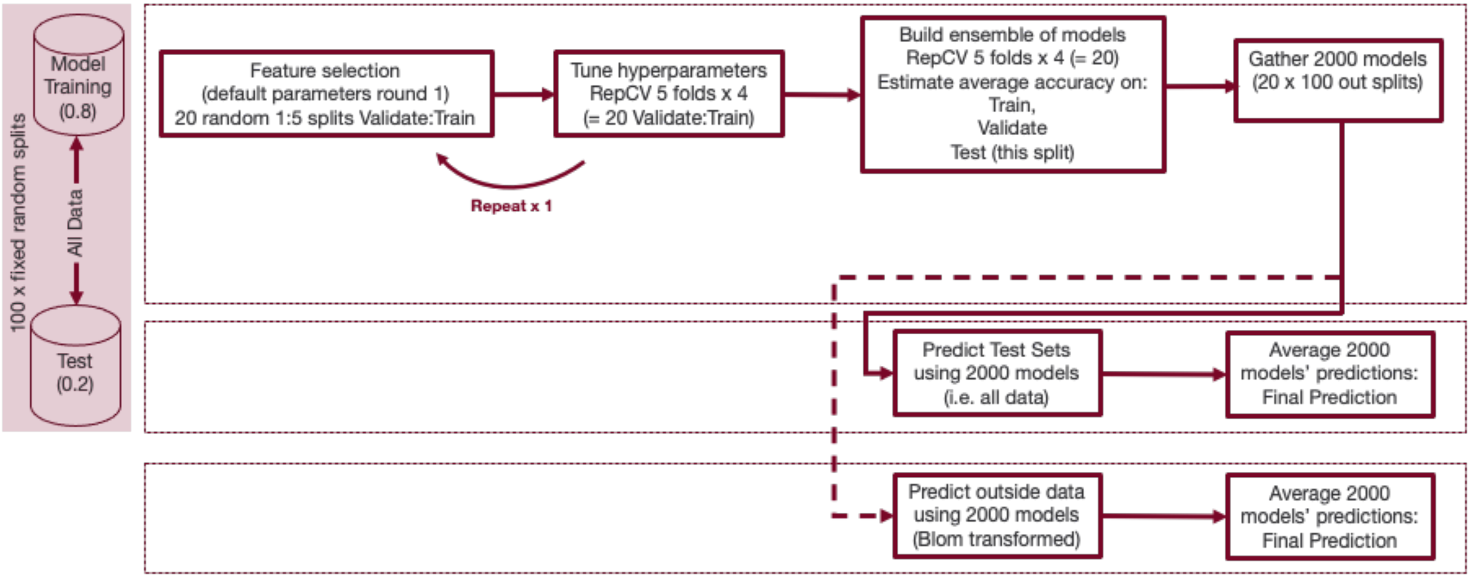

Supplemental Fig. S2 Generation of a 10 gene predictive gene expression signature. The 3433 leading-edge genes from a GSEA analysis of 21,693 functional gene sets were sorted by absolute significance score (|-log10(*P*) * $\log_2$ fold-change|) and the top 266 genes (by inflection point) were used as input for model training using boosted regression (XGBoost). A nested validation scheme was used. The 32 samples were divided 100 times, randomly, into an inner train-set and an outer hold-out set for 'testing'. The same sets were retained throughout the analysis. *Feature (gene) selection* was performed in two rounds. Each of the 100 inner train-sets were randomly divided into a training (80%) and a validation (20%) set and XGBoost run with default hyperparameters. This process was repeated 20 times and genes were ordered by their mean gain across all 2000 iterations (20 x 100). The top 50 genes were selected and the XGBoost hyperparameters optimized using repeated cross-validation (5 folds x 4 = 20). The top 50 genes were subject to a second round of feature selection analogous to the first except that the tuned hyperparameters were used and the top 10 genes extracted. The hyperparameters for the 10 genes were then also optimized. *The final models* were generated using both the top 10 and top 50 selected genes using repeated cross-validation (5 folds x 4 = 20) on each of the 100 inner sample sets. Performance metrics were gathered with each cross-validation test-set, and for predictions on the corresponding 100 hold-out test sets. These indicated that the 10-gene models were sufficient and performed better than the 50-gene models. The final model ('the gene signature') was the ensemble of these 2000, 10-gene, models. Each predicted sample was characterized as a responder or non-responder based upon the mode of the probability density distribution for that sample across all 2000 models

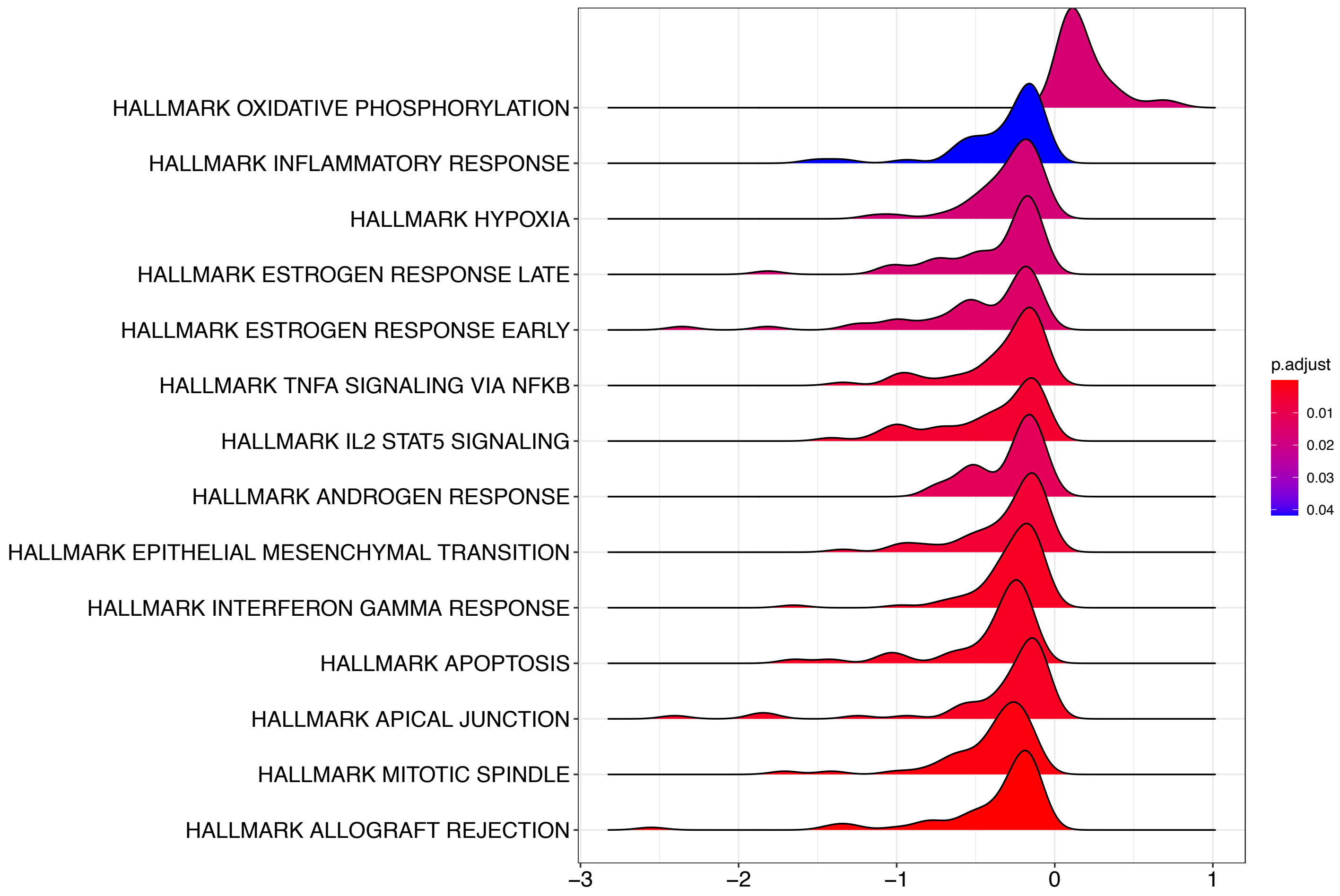

Supplemental Fig. S3 Gene Set Enrichment Analysis (GSEA), Hallmarks of Cancer.
Genes were ranked by their significance score (-log10(*P*) * $\log_2$ fold-change) after moderated t-tests (limma) using %PB-corrected and variance stabilized gene count data. Responders (BMR_CC) were enriched for expression of genes associated with OXPHOS whereas non-responders (no-BMR_CC) were enriched for expression of genes associated with inflammatory responses, steroidal hormone responses, apoptosis, and epithelial mesenchymal transition

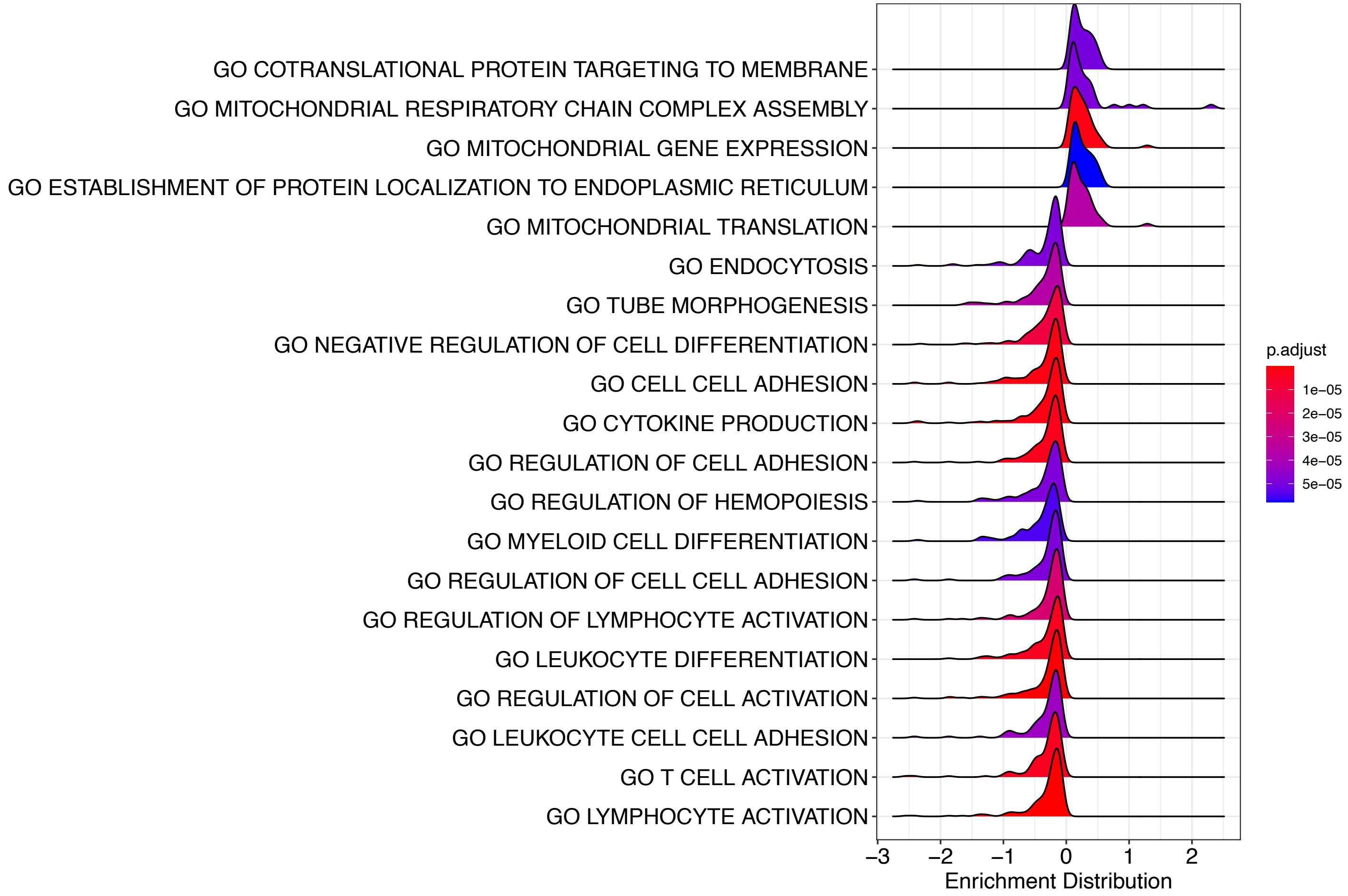

Supplemental Fig. S4 Gene Set Enrichment Analysis (GSEA), Gene Ontology Biological Processes.
Genes were ranked by their significance score (-log10(*P*) * $\log_2$ fold-change) after moderated t-tests (limma) using %PB-corrected and variance stabilized gene count data. Responders (BMR_CC) were enriched for expression of genes associated with OXPHOS and mitochondrial gene expression whereas non-responders (no-BMR_CC) were enriched for expression of genes associated with blood cell differentiation and immune cell activation

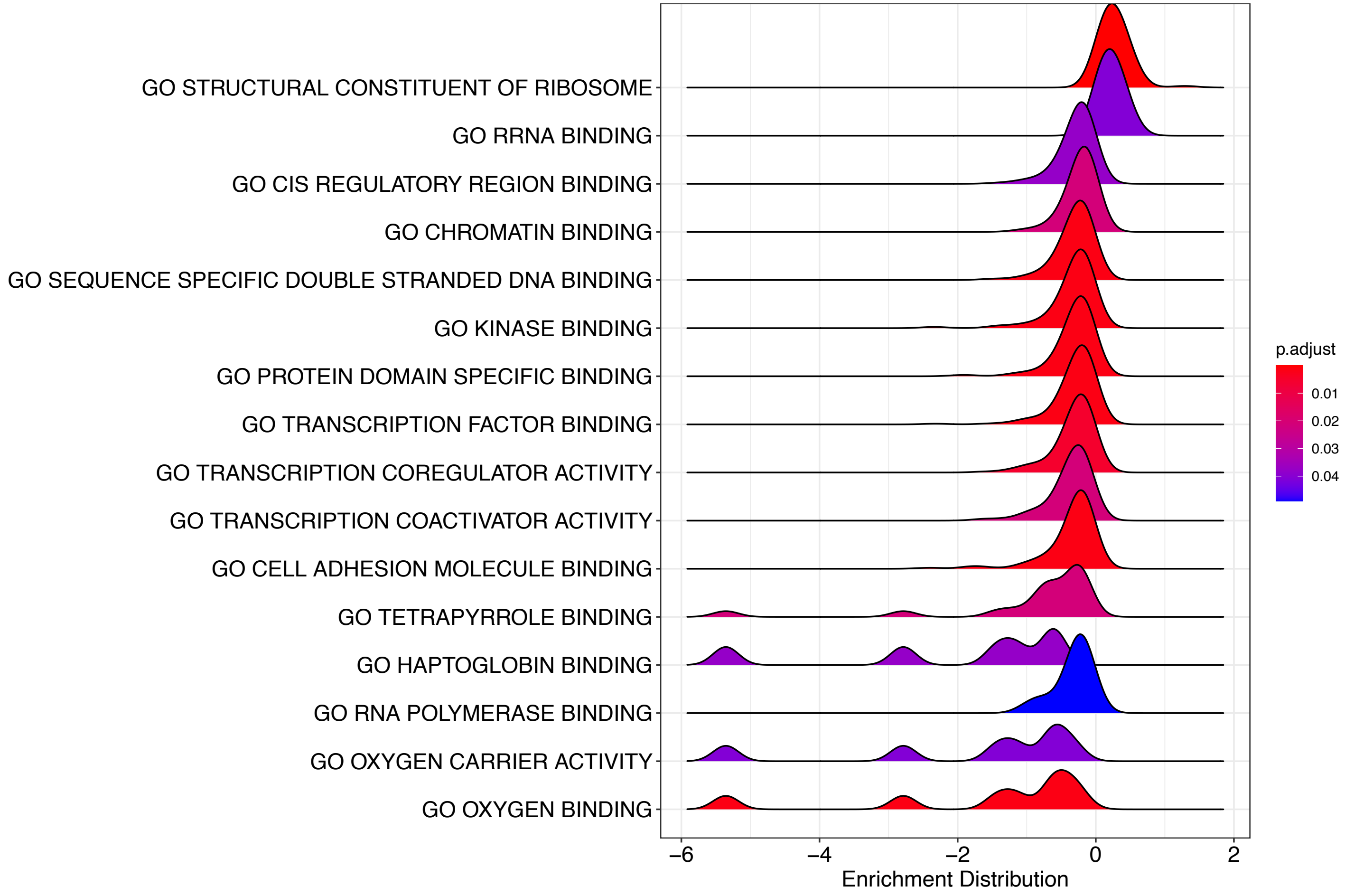

Supplemental Fig. S5 Gene Set Enrichment Analysis (GSEA), Gene Ontology Molecular Function.
Genes were ranked by their significance score (-log10(*P*) * $\log_2$ fold-change) after moderated t-tests (limma) using %PB-corrected and variance stabilized gene count data. Responders (BMR_CC) were enriched for expression of genes associated with ribosomal RNA gene expression, whereas non-responders (no-BMR_CC) were enriched for expression of genes associated with transcription factor binding and oxygen carriage (hemoglobins)

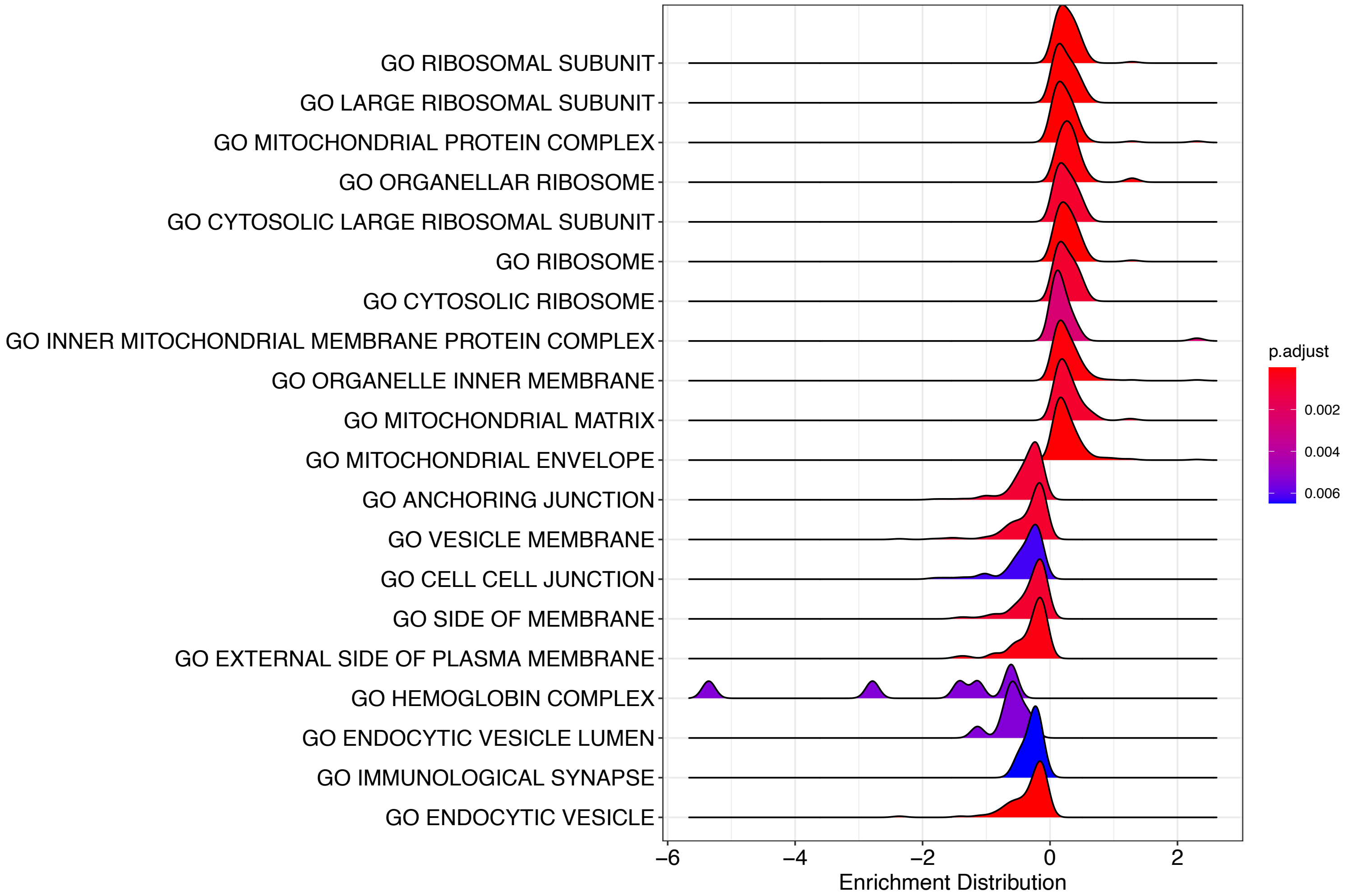

Supplemental Fig. S6 Gene Set Enrichment Analysis (GSEA), Gene Ontology Cellular Component.
Genes were ranked by their significance score (-log10(*P*) * $\log_2$ fold-change) after moderated t-tests (limma) using %PB-corrected and variance stabilized gene count data. Responders (BMR_CC) were enriched for expression of genes associated with mitochondrial and ribosomal RNA gene expression, whereas non-responders (no-BMR_CC) were enriched for expression of genes associated with cellular junctions, vesicles, and hemoglobins
